# Supplementary material for: Conditional chemoconnectomics (cCCTomics) as a strategy for efficient and conditional targeting of chemical transmission
Source: eLife. 2024 Apr 30;12:RP91927. doi: 10.7554/eLife.91927 (PMC11060718; doi:10.7554/eLife.91927)
Supplement: Figure 3—source data 3. [file elife-91927-fig3-data3.zip › Figure 3 source data3 part 2.zip!/Figure 3-Source data 3-part2/1060-9-CRISPResso_Report_q0_g4o36.zip!/CRISPResso_on_1060_12/1c.Alignment_barplot.pdf]

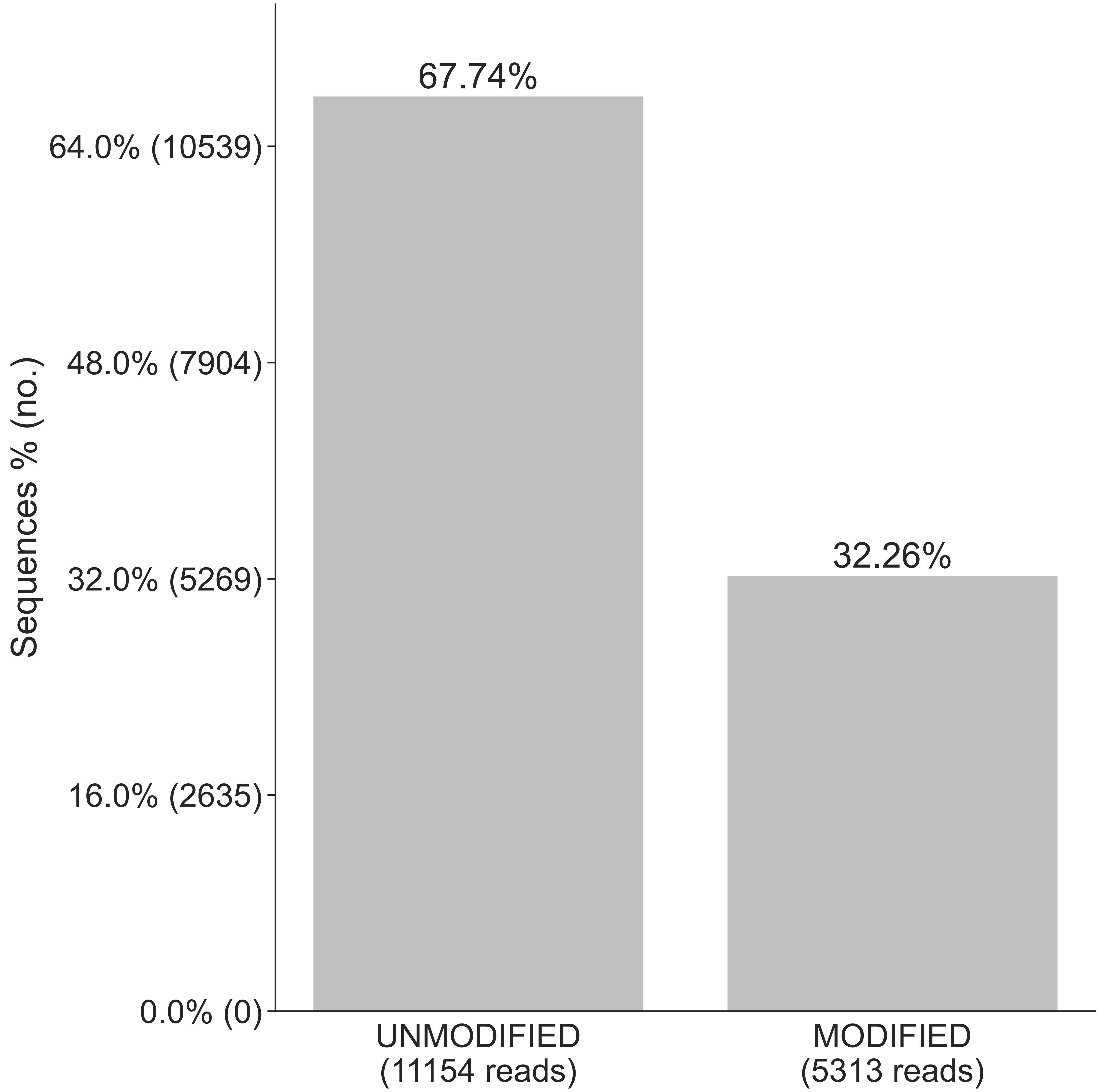
67.74%
32.26%
64.0% (10539)
48.0% (7904)
32.0% (5269)
16.0% (2635)
0.0% (0)
Sequences % (no.)
UNMODIFIED
(11154 reads)
MODIFIED
(5313 reads)
